# Pharmacy and Nursing Students’ Knowledge and Practices Concerning the Disposal of Unused and Expired Medicines in Kosovo

**DOI:** 10.3390/pharmacy10060145

**Published:** 2022-10-30

**Authors:** Selvete Shuleta-Qehaja, Nita Kelmendi

**Affiliations:** Department of Pharmacy, Campus College “Rezonanca”, Alma Mater Europaea, 10000 Prishtina, Kosovo

**Keywords:** pharmacy, nursing, students, medicines, pharmaceuticals, unused, expired, disposal

## Abstract

This descriptive cross-sectional study aimed to assess the knowledge and practices of pharmacy and nursing students at a medical college in Kosovo concerning unused and expired medications. A self-administered questionnaire was distributed to 500 randomly selected students of pharmacy (200 questionnaires) and nursing students (300 questionnaires). Overall, 336 returned the filled forms and the response rate was 67.2. SPSS version 26.0 was utilized for statistical analysis. The results showed that 89.2% of pharmacy students and 82.2% of nursing students check the expiration date of medications before purchasing them and a considerable number of students stated that they possess unused medicines at home (78.4% of pharmacy and 74% of nursing students). Regarding disposal practice, over 50% of both nursing and pharmacy students dispose of unused and expired medications in the trash. A small proportion of students returned unused or expired medicines to the pharmacy (11.4% of pharmacy students and 10.7% of nursing students return unused medications, whereas 14.4% of pharmacy respondents and 10.1% of nursing respondents reported returning expired medicines). There was a statistically significant difference in the ways pharmacy and nursing students purchase pharmaceuticals and in their opinions regarding institutions in charge of disposing of unused and expired medicines (*p* = 0.000). Students are aware of the detrimental effects improper disposal of unused and expired medicines has on the environment and public health, but lack information regarding the return of unused and expired medicines to the pharmacy. To change the existing practice the most appropriate method would be to add additional lectures on safe disposal practices into existing modules. It is recommended for involved stakeholders in Kosovo to organize training, seminars, and workshops for health professionals especially pharmacists and nurses, since they pass the information to patients/consumers as well as the government to make amendments to current legislation to ameliorate the returning procedures for patients/consumers in pharmacies.

## 1. Introduction

Medication consumption has been rising for decades because of improvements in clinical practice and a greater need for medicines to address age-related and chronic illnesses [1].

Due to various factors, such as a change in the patient’s condition, adverse events, or improvement in their condition, most of the time, recommended treatments go unused. Each of these contributes to a hazardous buildup of expired or unused medicines that must be disposed of [2], but the correct disposal practices may not always exist [3,4,5,6]. Unsafe drug disposal is escalating worldwide [7,8].

Kosovo is a developing country in southeastern Europe with a population of about 1.78 million [9]. In 2021, Kosovo’s GDP per capita was EUR 4486. In 2021, government health spending as a percentage of GDP was 3.4%, and government health spending per capita was EUR 151 [10]. Kosovo passed the Health Insurance Law in 2014, which makes mandatory health insurance available to all citizens. However, Kosovo has yet to put the Law into effect, leaving only private voluntary insurance costs. 

Two registered local pharmaceutical manufacturers produce generic medicines, and the majority of medicines are imported. Pharmacists or dispensing technicians are in charge of dispensing medications, and according to the publication in 2014 the most consumed medicines, are NSAIDs, and cardiovascular medicines, followed by antidiabetics and antibiotics [11]. 

Kosovo lacks a health insurance fund and relies on a limited essential drugs program procured and delivered directly through the Ministry of Health. Kosovo has no pricing regulation in the private sector, which accounts for approximately 85% of the total market. Nonetheless, the Ministry of Health purchases a limited number of essential drugs directly and distributes them for free in public health facilities [12].

No publicly funded outpatient medicines exist because no reimbursement system has been established, and patients must pay for all outpatient medication out of pocket. A published report in 2016 on pharmaceutical use in Kosovo showed that 79.7% of Kosovo residents procured medicines with a prescription and 63.5% without a prescription [13]. 

Accumulating unused and expired medicines in households usually results from excessive doctor prescribing [14] and/or poor patient adherence to prescribed medicines [15].

In general, unused and expired medicines in the EU countries should be returned to community pharmacies, which are obliged to collect unused medications from individual patients or consumers, or to a disposal site designated by the municipality.

According to the current legislation in Kosovo, the pharmacy is required to accept expired pharmaceuticals provided by individuals that were purchased there. The relevant Kosovo authority will pay for the costs incurred by the pharmacy as a result of the delivery of expired medications by individuals and the subsequent destruction of those medications by these governmental entities [16]. It is important to emphasize that there is currently little information available regarding the number and quantity of wasted medications wasted in Kosovo and that this could be hazardous to the environment and health.

In Kosovo, the Ministry of the Environment and Spatial Planning is responsible for issuing licenses to firms to dispose of pharmaceuticals. The Ministry of Health is accountable for support through the Pharmaceutical Inspectorate. Pharmacies are responsible for registering, identifying, and stocking products with an expiration date until they are disposed of by being classified as “unusable.” At the start of the disposal procedure, they must invite the Pharmaceutical Inspectorate to inspect the stock. The Ministry of the Environment and Spatial Planning requires the institution owning the waste to get environmental permission before disposing of expired pharmaceuticals. The waste owner enters into a contract with the permitted disposal site where the waste is destroyed. 

The Kosovo Law seeks to put EU waste management regulations into practice and provides a comprehensive framework for developing waste management, but its implementation is challenged by the lack of staff, poor institutional coordination, insufficient financing, and the lack of enforcement practices [17].

In developing countries, the safety and quality of healthcare are negatively impacted, and considerable financial resources are wasted since it is estimated that 60% of pharmaceuticals in public health facilities and 70% of medications in private facilities were prescribed and sold ineffectively [18,19]. Excessive prescription of medicines, the selling of unprescribed medications in community pharmacies, and the free distribution of medicines to the general public can all contribute to medication waste [20,21,22].

Additionally, the expenses related to disposing of unused and expired pharmaceuticals can be considered a financial burden. Numerous studies have been released indicating that the financial burden of pharmaceutical waste is becoming more well-acknowledged [23,24,25,26,27].

Managing this so-called “waste” has become a significant problem, according to research written by Alnahas et al. [28], because of the adverse effects that the accumulation of pharmaceutical waste has on the environment, the economy, social difficulties, and ethical concerns.

Active pharmaceutical substances have been found in the surface, the ground, and drinking water samples; these substances may be detrimental to aquatic life and ecosystems [29,30,31]. Patients must dispose of these correctly, perhaps by returning them to pharmacies or facilities to dispose of chemical waste. However, some patients might flush away or dispose of unnecessary prescriptions in the trash, jeopardizing the environment [32].

Patients interact with nurses and pharmacists more than any other medical staff, so they must be taught about safe disposal and storage practices at the young ages [32,33,34]. Pharmacy staff play a crucial role in the pharmaceutical supply chain and are well-positioned to reduce drug waste [26,35]. 

To our knowledge, this type of research is the first of its kind in Kosovo. The study aims to evaluate pharmacy and nursing students’ knowledge and practices regarding disposing unused and expired medications in Kosovo. 

## 2. Materials and Methods

This descriptive cross-sectional study took place at a medical college in Kosovo. The total number of active students registered in the pharmacy and nursing college database was 608 (221 pharmacy and 387 nursing, both BSc and MSc) at the beginning of the research. The questionnaire was distributed to 500 randomly selected students of pharmacy (200 questionnaires) and nursing students (300 questionnaires). The College Ethics Committee approved the study protocol (Protocol.No. AD-383/22). Students were given explanations about the objective of the study by the researchers. The self-administered questionnaire was delivered only to students, who gave verbal consent to participate in the study. They were also assured that the questionnaire was confidential and that the results would be presented anonymously. The research was performed from February 2022 until May 2022, and 336 students returned the filled forms. The overall response rate was 67.2%.

The questionnaire was changed and customized using pre-existing questionnaires from the published literature in the previous study [5,33,34,35,36,37,38,39,40]. The questionnaire was translated into the Albanian language. A pre-evaluation test of the translated version of the questionnaire was carried out by randomly choosing ten students from each group. They evaluated the translated version in the Albanian language of the questionnaire, which was slightly modified, and amended to suit students. The study did not include the students who took the pre-evaluation test. Using Cronbach’s alpha, a test of the questionnaire’s reliability revealed internal consistency reliability of α= 0.705.

SPSS statistics (version 26.0, SPSS Inc., Chicago, IL, USA, accessed on 20 May 2022) was used to analyze the collected data. Since questions are categorical variables, their proportions and percentages have been determined. In addition, the associations between variables were determined by performing chi-square tests. A *p*-value < 0.05 was considered a statistically significant difference in all analyses. 

## 3. Results

The following table (Table 1) displays the study’s participants’ demographic information. There were 336 returned questionnaires, 169 of which were from nursing students and 167 from pharmacy students. Female students made up 79.8% of the respondents. The majority of the students ranged in age from 18 to 22 years old. Most nursing students (45.6%) and pharmacy students (43.1%) who responded are in their third year of bachelor’s studies, respectively.

Table 2 presents information on how respondents acquire medications. It shows that 78.4% of pharmacy students and 97.6% of nursing students stated that they buy medicines using a prescription, whereas 33.5% of pharmacy students and 12.4% of nursing students declared to purchase medication without a prescription as recommended by the pharmacist. In both questions, it was a significant difference (*p* = 0.000) between the two groups of responding students. A high percentage of both respondent groups stated that they do not buy medicines based on the advice of a relative/friend and neither receive medicines from a friend /relative. 

Students are generally supportive of checking the expiration date before purchasing medications. Overall, 82.2% of nursing students and 89.2% of pharmacy students gave positive answers. However, a considerable number of students (78.4% of pharmacy students and 74% of nursing students) admitted to having unused medications at home. Over 50% of nursing and pharmacy students dispose of unused medications in the trash, as shown in Table 3. It is concerning as 73.1% of pharmacy students and 79.3% of nursing students reported that they do not return unused medicines to the pharmacy. Similar percentages of responses were received regarding the disposal practice of expired medications. In total, 76% of pharmacy students and 82.8% of nursing students stated that they do not return expired medications to the pharmacy. These results may indicate that they are not informed about the existing process of returning unused and expired medicines.

Respondents’ opinions on institutions in charge of unused or expired medications are displayed in Table 4. The chi-square test results show significant differences (*p* = 0.000) in nursing and pharmacy students’ perspectives on the Ministry of the Environment and Spatial Planning duties. 

In total, 95.2% of pharmacy and 97% of nursing students concurred that inappropriate medicine disposal could influence the environment and public health, as presented in Table 5.

## 4. Discussion

Our results suggest that pharmacy and nursing students are uninformed of safe disposal options for unused and expired pharmaceuticals. Similar to the study by Ali [41], Auta et al. [42] and by Shakib et al. [43]. According to similar surveys, most academics, including pharmacy students, dispose of unused or expired medications in their regular trash as their preferred disposal method, the lack of understanding of how to manage these pharmaceutical waste products may explain this practice [44,45]. 

Raja et al. [5] found that 92.8% of participants checked the expiration dates of pharmaceuticals and our findings are similar but better than those of Bashatah and Wajid’s [40] study, which found that only 57.4% of pharmacy students and 53.4% of nursing students checked the expiration dates of medications.

The results of our survey indicate that pharmacy and nursing students practice incorrect disposal by throwing expired medicines in the trash. Similar to our findings, 34% of pharmacy students who participated in a study by Shakib et al. [43] indicated they frequently threw their remaining medicines in the trash. When asked if they threw away unused prescriptions in the garbage, 53.1% of the pharmacists in the Atia [44] survey stated that they did. According to the research report from Bashatah and Wajid [40]^,^ 74.2% of nursing and 68.3% of pharmacy students disposed of expired prescriptions in the regular trash. The results of our study agree with Raja et al. [5], who discovered that 72% of respondents disposed of expired prescription drugs. Aditya and Singh [45] found that 94% of participants in a related study of dental students threw away any leftover medication in the trash at home. Whether the subjects are members of the general public or medical professionals, this behavior has been observed globally [5,46]. In the study by Viana et al. [47], 48.4% of the participating pharmacy students said they disposed of their medications in domestic garbage. Similarly, 65% of medical professionals who answered a survey in a study by Swaroop et al. [48] in 2015 stated that they throw away unwanted medications in the trash. In a study by Kaur and Bansal [49], the most common method of medicines disposal among medical personnel was household trash (74.8%).

In our study, 52.1% of pharmacy students declared to dispose of unwanted medications in the trash, compared to 37% of pharmacy students in the study by Bashatah and Wajid [40].

According to our study’s results, just a small portion of both groups returned unused or expired medications to the pharmacy. Our findings align with a study published by Alhomoud et al. [50], in which 15% of pharmacy students reported returning unused medications to the pharmacy. In Aditya’s study [45], just 3% of respondents stated returning unused or expired medicines to the pharmacy.

More than half of respondents (54.5% of pharmacy students and 55% of nursing students) think that disposing of unused and expired medications is not the pharmacist’s responsibility. The majority (64.1% of pharmacy students and 67.5% of nursing students) responded that it is the Ministry of Health’s responsibility. However, just 17.2% of nursing students agree with 35.9% of pharmacy students who claim it is the Ministry of the Environment and Spatial Planning’s responsibility.

Both pharmacy and nursing students knew that inappropriate disposal of unused and expired medicines impacts negatively public health and the environment. The results of our study are comparable to those of other studies that have been published involving both student groups and the general public [5,36,38,43,51,52].

To promote awareness and education on the safe disposal of medicines, it is recommended for involved stakeholders in Kosovo to organize training, seminars, and workshops for health professionals especially for pharmacists and nurses, since they pass the information to patients/consumers. It is evident that there is a low percentage of the budget dedicated to health, and the Ministry of Health already faces many challenges. Still, this could be accomplished with the support of international institutions present in Kosovo whose programs are directed towards the environment and climate change. 

Another issue that has been noticed is that by the current legislation, unused and expired medicines can be returned only to pharmacies where the consumer/patient bought the medicine by presenting the invoice, which might cause impracticalities for consumers/patients. The regulation should be such to facilitate the return of medicines without additional administrative burdens; thus, an amendment to this regulation is recommended so that it can be implemented in practice.

There are several limitations to this study. Our research only included students of pharmacy and nursing from one college. To generalize the results, future research involving students from both public and private educational medical institutions is recommended. It is also suggested that practicing pharmacists and nurses be included. The limitation is related to the questionnaire which was changed and customized according to the circumstances in Kosovo using pre-existing questionnaires from the published literature. However, it remains to prepare a comprehensive questionnaire in future studies.

## 5. Conclusions

The findings of our study show that nursing and pharmacy students’ current practice and knowledge regarding the disposal of unused and expired medications were insufficient. They are aware of the negative implications of incorrect disposal of unused and expired medicines for the environment and public health. To enhance the knowledge and change their existing practice the most appropriate method would be to add additional lectures on safe disposal practices into existing modules. Additionally, the government should organize training, seminars, and workshops for health professionals about the proper methods of drug disposal.

## Figures and Tables

**Table 1:** Demographic characteristics of respondents (*n* = 336).

Respondents		Pharmacy (*n* = 167)	Nursing (*n* = 169)
		N (%)	N (%)
Gender	Male	38 (22.8)	30 (17.8)
	Female	129 (77.2)	139 (82.2)
Age	18–22	122 (71.1)	145 (85.8)
	23–26	20 (12)	15 (8.9)
	27–30	7 (4.2)	4 (2.4)
	>30	18 (10.8)	5 (3)
Year of Studies	BSc–Year 1	38 (22.8)	31 (18.3)
	BSc–Year 2	14 (8.4)	57 (33.7)
	BSc–Year 3	72 (43.1)	77 (45.6)
	MSc–Year 1	27 (16.2)	0 (0)
	MSc–Year 2	16 (9.6)	4 (2.4)

Note: *n*—number; Frequency—N; percentage—%.

**Table 2:** Responses of students regarding the ways of procuring medicines.

How Do You Purchase Medicines?			
	Pharmacy (*n* = 167)	Nursing (*n* = 169)	
*Responses*	N (%)	N (%)	*p*-Value
With prescription			
Yes	131 (78.4)	165 (97.6)	0.000
No	36 (21.6)	4 (2.4)	
Without prescription (OTC)			
Yes	56 (33.5)	21 (12.4)	0.000
No	111 (66.5)	148 (87.6)	
On the advice of a friend/relative			
Yes	34 (20.4)	22 (13)	0.048
No	133 (79.6)	147 (87)	
Receive medicines from a friend/relative			
Yes	19 (11.4)	16 (9.5)	0.347
No	148 (88.6)	153 (90.5)	

Note: number—n; frequency—N; percentage—%

**Table 3:** Responses of students concerning the disposal practice of unused and expired medicines.

	Pharmacy (*n*=167)	Nursing (*n*=169)	
	N (%)	N (%)	*p*-value
What do you do with unused medicines?			
*Responses*			
Throw it in the garbage			0.252
Yes	87 (52.1)	95 (56.2)	
No	53 (31.7)	57 (33.7)	
I do not know	27 (16.2)	17 (10.1)	
Flush in the sink/toilet			0.1
Yes	15 (9)	10 (5.9)	
No	121 (72.5)	139 (82.2)	
I do not know	31 (18.6)	20 (11.8)	
Return them to the pharmacy			0.292
Yes	19 (11.4)	18 (10.7)	
No	122 (73.1)	134 (79.3)	
I do not know	26 (15.6)	17 (10.1)	
Give to friends/relatives unused medicines			0.13
Yes	26 (15.6)	35 (20.7)	
No	113 (67.7)	117 (69.2)	
I do not know	28 (16.8)	17 (10.1)	
What do you do with expired medicines?			
*Responses*			
Throw it in the garbage			0.835
Yes	117 (70.1)	123 (72.8)	
No	37 (22.2)	35 (20.7)	
I do not know	13 (7.8)	11 (6.5)	
Flush in the sink/toilet			0.335
Yes	22 (13.2)	16 (9.5)	
No	132 (79)	144 (85.2)	
I do not know	13 (7.8)	9 (5.3)	
Burn them			0.145
Yes	10 (6)	5 (3)	
No	143 (85.6)	156 (92.3)	
I do not know	14 (8.4)	8 (4.7)	
Return them to the pharmacy			0.303
Yes	24 (14.4)	17 (10.1)	
No	127 (76)	140 (82.8)	
I do not know	16 (9.6)	12 (7.1)	

Note: number—n; Frequency—N; percentage—%.

**Table 4:** Responses of students concerning their knowledge of the responsibilities of different parties for the disposal of unused and expired medicines.

Who is Responsible for the Disposal of Unused and Expired Medicines in Kosovo?			
	Pharmacy (*n* = 167)	Nursing (*n* = 169)	
*Responses*	N (%)	N (%)	*p*-value
The pharmacist			
Yes	50 (29.9)	60 (35.5)	0.192
No	91 (54.5)	93 (55)	
I do not know	26 (15.6)	16 (9.5)	
The Ministry of Health			
Yes	107 (64.1)	114 (67.5)	0.332
No	39 (23.4)	42 (24.9)	
I do not know	21 (12.6)	13 (7.7)	
Ministry of the Environment and Spatial Planning			
Yes	60 (35.9)	29 (17.2)	0.000
No	80 (47.9)	121 (71.6)	
I do not know	27 (16.2)	19 (11.2)	

Note: number—n; Frequency—N; percentage—%.

**Table 5:** Responses of Students Regarding Their Knowledge of the Impact of Incorrect Disposal of Unused and Expired Medicines on the Environment and Health.

Does inappropriate disposal of unused and expired medicines affect health and the environment?			
	Pharmacy (*n*=167)	Nursing (*n*=169)	
*Responses*	N (%)	N (%)	*p*-value
Yes	159 (95.2)	164 (97)	0.062
No	5 (3)	0 (0)	
I do not know	3 (1.8)	5 (3)	

Note: number—n; Frequency—N; percentage—%.

## Data Availability

The datasets generated during and/or analyzed during this study are not publicly available due to the use of anonymous survey data; however, datasets are available from the corresponding author on reasonable request.
